# Leveraging Novel Integrated Single-Cell Analyses to Define HIV-1 Latency Reversal

**DOI:** 10.3390/v13071197

**Published:** 2021-06-22

**Authors:** Suhui Zhao, Athe Tsibris

**Affiliations:** Brigham and Women’s Hospital, Harvard Medical School, Boston, MA 02139, USA; szhao13@bwh.harvard.edu

**Keywords:** HIV latency, virus reservoir, single-cell RNA-seq, single-cell ATAC-seq, CITE-seq

## Abstract

While suppressive antiretroviral therapy can effectively limit HIV-1 replication and evolution, it leaves behind a residual pool of integrated viral genomes that persist in a state of reversible nonproductive infection, referred to as the HIV-1 reservoir. HIV-1 infection models were established to investigate HIV-1 latency and its reversal; recent work began to probe the dynamics of HIV-1 latency reversal at single-cell resolution. Signals that establish HIV-1 latency and govern its reactivation are complex and may not be completely resolved at the cellular and regulatory levels by the aggregated measurements of bulk cellular-sequencing methods. High-throughput single-cell technologies that characterize and quantify changes to the epigenome, transcriptome, and proteome continue to rapidly evolve. Combinations of single-cell techniques, in conjunction with novel computational approaches to analyze these data, were developed and provide an opportunity to improve the resolution of the heterogeneity that may exist in HIV-1 reactivation. In this review, we summarize the published single-cell HIV-1 transcriptomic work and explore how cutting-edge advances in single-cell techniques and integrative data-analysis tools may be leveraged to define the mechanisms that control the reversal of HIV-1 latency.

## 1. Introduction

HIV-1 persists in CD4^+^ T cells and rebounds after antiretroviral therapy (ART) is interrupted or stopped. Viral latency is defined as a state of reversible nonproductive infection that, in the case of HIV-1, is associated with the use of suppressive ART [1]. Previous work investigated which proportion of integrated sequences are intact, and thereby may contribute to the replication-competent HIV-1 reservoir [2,3,4,5]. The vast majority, greater than 90%, of integrated proviruses are defective, accumulate during acute HIV-1 infection, and cannot support viral replication [2,4]. However, these “defective” proviruses may still retain HIV-1 transcriptional and translation activity that is relevant to eradication efforts [6,7].

Cells harboring latent HIV-1 are extremely rare, numbering in the range of 10^0^–10^3^ per million CD4^+^ T cells in peripheral blood during treated suppressed infection, and may be heterogenous [8,9,10,11]. As few as 100 CD4+ T cells harbor intact HIV-1 proviruses that may contribute to rapid HIV-1 rebound after ART is stopped [2,4,12]. Cells that comprise the HIV-1 reservoir express no known distinguishing markers, making them a challenge to identify [2,7,13,14,15,16,17,18]. Several approaches were developed to identify cells harboring HIV-1 DNA and measure the frequencies, size, and phenotypes of the HIV-1 reservoir in ART-suppressed individuals. The tat/rev-induced limiting dilution assay (TILDA) can measure multiply spliced HIV-1 transcripts after stimulation and has the advantage of requiring fewer cells than other methods [19,20,21], while the quantitative viral outgrowth assay (QVOA), a gold standard of estimation of the size of HIV-1 reservoir, quantifies replication-competent proviruses [22,23,24,25]. Even the gold standard has limitations: repeated rounds of activation change the result, and a newer QVOA that preferentially differentiates T cells to an effector memory phenotype, dQVOA, may be more accurate [26]. The intact proviral DNA assay (IPDA) may offer an accurate and scalable alternative to the more labor-intensive QVOA [12,27]. Full-length sequencing of the HIV-1 genome can quantify intact noninduced genomes of HIV-1 [3,4]. Cells expressing the HIV-1 capsid protein, p24, that is produced during viral reactivation can be quantified by flow cytometry [28,29,30]. These approaches should be viewed as complementary; sequencing cannot determine translation or replication competence, and flow cytometry cannot assess proviral deletions and/or mutations. 

The rarity of latently HIV-infected cells in patients makes their reactivation a challenge to study at the single-cell level. To address this difficulty, in vitro and ex vivo models were developed to investigate HIV-1 latency, each with their own unique sets of advantages and disadvantages. In vitro models can provide sufficient numbers of cells for analyses, but may not recapitulate latency in vivo, and are rarely truly latent [31]. Primary ex vivo models that use CD4^+^ T cells are widely used in the investigation of HIV-1 latency and latency reversal [24,32,33,34,35,36]. 

**Figure 1:**
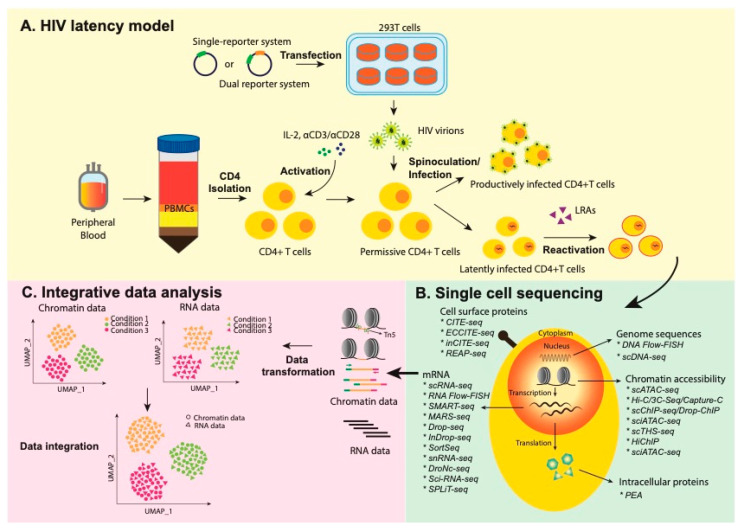
Schematic of integrative analysis of HIV-1 latency. (**A**) Establishment of HIV-1 latency in model systems that use primary human CD4^+^ T lymphocytes. Generally, cells are activated, infected by reporter HIV-1 constructs, and allowed for returning to a quiescent state prior to reactivation with pharmacologic latency reversal agents (LRA). (**B**) Summary of single-cell technologies to characterize transcriptome, proteome, and regulome. (**C**) Integration approaches to single-cell datasets that assess transcription and chromatin accessibility. Tn5 transposases, coupled with DNA adapters, fragment and tag accessible genomic DNA. Resulting fragments are amplified and sequenced to generate chromatin accessibility data.

The availability of high-resolution single-cell RNA sequencing (scRNA-seq) in combination with novel technologies that assess the epigenome and proteome, such as the assay for transposase-accessible chromatin using sequencing (scATAC-seq) and Cellular Indexing of Transcriptomes and Epitopes by Sequencing (CITE-seq), provides a unique opportunity to study the heterogeneity of HIV-1 latency reversal, and more precisely define the mechanisms that control HIV-1 transcriptional reactivation (Figure 1). In this review, we summarize current approaches to leverage single-cell sequencing technologies to study HIV-1 latency. We review downstream integrative-analysis approaches to identify new strategies that may shed light on the complexities of HIV-1 latency and its reactivation.

## 2. Single-Cell Studies of HIV-1 Latency

Major contributions to our understanding of the HIV-1 reservoir and its latency come from the study of cells in bulk. This is true for how the reservoir is established, how it is maintained, and how its latency may be pharmacologically reversed [4,22,23,24,37,38,39,40]. Based on these important discoveries, latency-reversal agents (LRA) were advanced into clinical studies but have, to date, underwhelmed [41,42,43,44]. The general concept of latency reversal is that, during suppressive ART that prevents viral replication, HIV-1 proviruses are induced to transcribe and translate viral proteins, “marking” the cell for subsequent immune-system clearance. However, HIV-1 latency is not always effectively reversed and total reservoir size in clinical trials post-LRA exposure remains unchanged. Mathematical-modeling studies suggest that a 3–4 log_10_ reduction in HIV-1 reservoir size may be required to achieve a reasonable probability of viral eradication; an empirical study in stem-cell transplant participants supports this idea [45,46].

To better understand the relationship between HIV-1 latency reversal and CD4^+^ T cells, recent studies have focused on the mechanisms that define latency at single-cell resolution (Table 1). More insight into cell-to-cell variations that predict effective latency reversal may identify key nodes or checkpoints in cell function and further efforts to improve LRA performance. In general, the purpose of single-cell approaches is to more clearly resolve important cellular and regulatory signals that may be obscured in the aggregated and averaged data generated by bulk cell analyses. There are reasons to believe that the heterogeneity of the HIV-1 reservoir may justify single-cell approaches to study viral latency. 

Treatment with LRAs leads to the transcriptional reactivation of only a small subset of total proviruses, even with more than one round of stimulation [4,47,48]. Researchers hypothesized that heterogeneity in the HIV-1 integration site, CD4^+^ T-cell subsets, and their activation state, epigenetic modifications, and stochastic transcriptional noise [49,50] may play a role in viral latency and justify experiments at the single-cell level that characterize transcriptional diversity and resolve heterogeneity in regulatory mechanisms. HIV-1 may integrate into euchromatin, heterochromatin, and gene “deserts”, be present as intact replication-competent provirus, defective transcriptionally silent provirus, or replication-defective provirus that is still capable of some viral protein translation [7,16]. This heterogeneity extends to T-cell subsets that are infected and their relative state of rest or activation, all of which can be studied with greater granularity and detail at single-cell resolution [39,51,52]. Studies published in the past 3 years, discussed below, probed these important questions in a primary HIV-1 latency model and in ex vivo samples from treated suppressed participants with HIV-1. Key experimental details of these studies are summarized in Table 1.

Due to the rarity of latent cells in ART-treated virologically suppressed participants with HIV-1, most studies leverage lab-adapted or primary-latency models, the scope of which is beyond this review. Two studies used a well-described primary-latency model to investigate how the host transcriptional program influences HIV-1 latency at the single-cell level [50,53]. In this model, CD4^+^ T cells are activated, infected with a GFP-expressing reference HIV-1 strain NL4-3, and then cultured for 8–12 weeks on H80 cells. H80 cells are a lab-adapted adherent glioma cell line that rescues T cells from death and reduces their activation, as defined by HLA-DR and CD25 expression [54]. More recent work identified TGF-β and IL-8 as cytokines secreted by H80 cells that play a role in the induction of T-cell quiescence [34]. After 12 weeks of coculture, these CD4^+^ T cells display a heterogeneous range of GFP expression. 

To understand heterogeneity in HIV-1 latency and its reversal, HIV-1-infected CD4^+^ T-cell populations after 8 weeks of H80 cell coculture were incubated in the presence or absence of vorinostat (VOR), a histone deacetylase inhibitor, for 24 h or tetrameric anti-CD3/anti-CD28 antibody complexes (TCR) for 48 h [53]. A total of 224 cells, namely, 43 untreated, 90 VOR-treated, and 91 TCR-treated, were then subjected to Smart-seq single-cell analyses. Principal-component analysis (PCA) identified two main clusters: a well-demarcated Cluster 1 and a larger Cluster 2. Each cluster contained cells from all three treatment conditions, suggesting some commonality in transcription that did not depend on drug exposure. CD4^+^ T cells in this model therefore generally exist in at least two transcriptional states. Visually, TCR-treated cells in Cluster 2 segregated from untreated and VOR-treated cells, possibly indicating a TCR activation-specific effect on transcription. The two main clusters significantly differed in GFP intensity, as assessed by fluorescent microscopy, and suggested that Cluster 1 corresponded to noninduced cell phenotypes, and Cluster 2 to inducible cells. However, GFP fluorescence in VOR-treated cells was not observed. HIV-1 transcript levels were higher in Cluster 2 across treatment conditions, suggesting that perhaps a mixture of productively and latently HIV-1-infected cells were included in the analyses. This identifies a potential source of confounding: CD4^+^ T cells may have expressed HIV-1 transcripts either because they were productively infected pre-stimulation, or because HIV-1 was, in fact, latent and was reactivated by TCR-mediated signaling. A total of 134 differentially expressed genes were identified that distinguished Clusters 1 and 2, approximately half of which were ribosomal, and half were enriched in pathways specific for metabolism, translation, electron transport, splicing, and HIV-1 infection. To confirm and extend this signature, analysis of a total of 77 resting CD4^+^ T cells, defined as CD25-CD69-HLA-DR-, isolated from two treated suppressed participants with HIV-1 identified similar differential expression of these 134 genes. Whether this signature is a function of T-cell biology or the presence of integrated HIV-1 and its reactivation remains an important open question.

In a study later that same year, the transcriptomes of CD4^+^ T cells from three donors, infected ex vivo with HIV-1 and maintained on H80 cells for 6–12 weeks, were assessed using a 3′ 10× Genomics sequencing approach [50]. Dimensionality reduction and visualization in t-distributed stochastic neighbor embedding (tSNE) plots of data generated from 4206 cells demonstrated an expected clustering by donor. To probe the association of host transcriptome with HIV-1 latency, differences in gene expression were assessed between cells with and without measurable GFP transcripts. Cells with latent GFP expressed a specific set of cellular genes, marked by higher CCR7, CD27, and SELL transcripts, when compared to highly GFP-expressing cells that demonstrated greater levels of IL2RA, HLA-DRA, CD38, TNFRSF4, and TNFRSF18 transcripts. Cells enriched for transcripts in cell death or survival and proliferation pathways, as identified in Ingenuity Pathway Analysis, were more likely to express GFP. After cell activation through T-cell receptor signaling with anti-CD3/anti-CD28 beads, however, it was GFP-cells that demonstrated greater proliferative capacity, preferentially expanding naïve and central memory T-cell subsets. When comparing these two published reports that used the H80 latency model, some similarities in gene expression were observed but also many differences that may in part be due to technical variations in the precise methodologies that the two groups used. 

To explore the gene-expression program associated with ex vivo HIV-1 latency reversal, investigators developed a method to isolate CD4^+^ T cells that express HIV-1 envelope (Env) after reactivation [55]. Purified CD4^+^ T cells isolated from 10 treated suppressed participants with HIV-1 were cultured for 36 h in PBMC-conditioned media that contained phytohemagglutinin (PHA), IL-2, and a pan-caspase inhibitor. Incubation with a cocktail of three broadly neutralizing antibodies (bnAb), which targeted the CD4 binding site and the V2 and V3 loops, enriched for cells that expressed HIV-1 mRNA. Env-expressing cells from three participants that coexpressed intracellular HIV-1 Gag underwent scRNAseq; a total of 85 such cells were sequenced to an average depth of 1500 expressed genes per cell. Approximately 4% of reads in Env^+^Gag^+^ cells mapped to HIV-1 and, when combined with T-cell receptor-sequencing analysis, strongly suggested a clonal origin of these reactivated cells. Using a Seurat analysis approach that included comparator unfractionated PHA-activated cells and unrelated cells productively infected with the YU2 reference strain, a set of differentially expressed genes in PHA-activated, HIV-1 Env- and Gag-expressing cells were identified [56]. These genes were enriched in processes mostly related to immune-system function.

More recently, a method known as SortSeq was developed and used to assess cellular transcription during latency reversal [57] (Table 1). The approach incubates resting CD4^+^ T cells, defined as CD25^−^CD69^−^HLA-DR^−^, isolated from participants with HIV-1 in the presence of phorbol 12-myristate 13-acetate (PMA) and ionomycin for 16 h. Cells are then fixed, permeabilized, and incubated with fluorescently labeled probes that are designed to bind the 5′ and 3′ regions of HIV-1 mRNA molecules. Probe-positive cells are isolated by flow cytometry and sequenced by SMART-seq. In this study, a total of 48 cells were analyzed from 14 treated virologically suppressed participants with HIV-1 (mean 3.4 cells, median 2 cells per participant). Differentially expressed genes in these SortSeq-identified cells were enriched for RNA binding proteins and pathways related to RNA processing and immune-system function.

Taken together, these studies provide important insights into the transcriptional changes that associate with HIV-1 latency reversal. To advance the field further and facilitate comparisons across studies, subsequent experiments should verify the HIV-1 latency state of the cell prior to reactivation and downstream analyses. To evaluate the scientific rigor of primary latency model systems, the markers used to define latency, fluorescent or viral, should be quantified at the protein and RNA level and compared. Appropriate HIV-uninfected comparator conditions should be included and reactivation conditions should ideally be standardized. Corroborating the findings of these early pioneering single-cell studies should be a priority, along with further mechanism-driven work to determine how the identified pathways more specifically impact viral latency.

**Figure 2:**
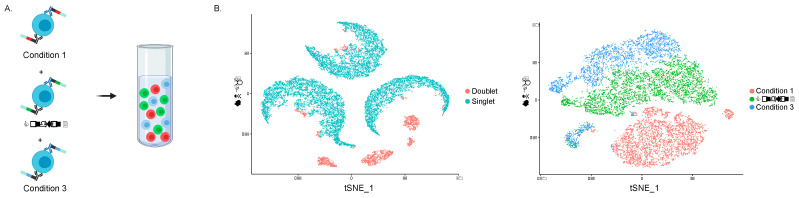
Cell hashing. To reduce batch effects and technical variations, cells from different conditions can be combined into one scRNAseq experiment and later demultiplexed. (**A**) Cells are labelled with identical antibodies to ubiquitous surface proteins bound to hashtag oligonucleotides (HTO) that comprise a common PCR handle (black) and poly-A tail (light blue) on either side of a 15-nucleotide sequence (red, green, or blue) that is unique to each experimental condition. (**B**) Cells that are positive for more than one HTO are annotated as doublets, and cells negative for all HTO are assumed to be empty droplets (ambient RNA). Singlets are then extracted, and tSNE plots generated from these hashtag count values, effectively identifying and clustering cells from each condition contained in the mixture.

To mitigate batch effects in experimental designs, and more accurately identify and remove cell multiplets, cell hashing can combine comparator conditions into one sequencing run [58,59,60] (Figure 2). Cell hashing is a process that labels cells with sequence-tagged monoclonal antibodies to common surface proteins such as the β3 Na^+^/K^+^ ATPase and β2-microglobulin, permits the unambiguous identification of cell multiplets, and can assign cells from different conditions back to their original sample.

Disentangling the transcriptome associated with T-cell activation biology from any changes specifically associated with the presence of integrated HIV-1 genomes should be prioritized, focusing on total CD4^+^ T-cell populations and participant-to-participant variations in transcriptome. While cost remains an issue in single-cell experimental design, technological advances permit the aggregation of experimental data across groups and platforms, increasing their power and value [56]. Sequencing a larger number of cells improves the reproducibility of results, a process made simpler with the wider availability of droplet-based single-cell sequencing methods to increase throughput. Importantly, future work should extend to myeloid cells, a potential HIV-1 reservoir, and rapidly developing spatial transcriptomics approaches to study latency in tissue.

To confirm findings from single-cell HIV-1 analyses, orthogonal methods that leverage more classical testing approaches should be used. As the current literature highlights, there is not always commonality in the findings across single-cell studies of HIV-1 latency. While it is perhaps premature to suggest a standardized confirmatory approach, classical testing helps to disentangle which findings relate more to the used latency system or the employed precise experimental approach, and which elucidate the fundamental biology of HIV-1 latency reversal.

## 3. Multimodal Profiling of HIV-1 Latency

Single-cell transcriptomics can characterize the cellular heterogeneity that is present in primary HIV-1 latency model systems and in samples from patients with HIV-1. To define the regulatory mechanisms that govern transcription and its downstream translation, single-cell RNA sequencing may be combined with assessments of the epigenome and proteome. Combining -omics approaches may help answer a more fundamental question: at which level—DNA, RNA, or protein—is the latency-reversal program encoded, if one exists? Multimodal profiling offers advantages during the dynamic cellular-transition states associated with viral latency reversal, when the correlation between transcript and protein levels may weaken [61]. We summarize these modalities and discuss how they may be applied to the study of HIV-1 latency reversal. Important work that incorporates RNA and protein measurements to study HIV-1 latency at single-cell resolution was developed, but it does not have the throughput of droplet-based single-cell profiling and is not enumerated in this review [12,44,51,62,63,64,65]. 

With combinatorial labelling and barcode approaches, single-cell sequencing may be widely applied to analyze epigenomic [66,67], transcriptomics [68], chromatin accessibility [69,70,71,72,73], cell-surface proteins [74,75], and chromosomal conformation [76,77]. A variety of single-cell sequencing approaches were developed to characterize mRNA, including Drop-seq, InDrop, Smart-seq, MARS-seq, 10X Genomics, SPLiT-seq, sci-RNA-seq; surface-protein identification and cell phenotyping using CITE-seq, REAP-seq, FACS; and chromatin-accessibility measurements with scATAC-seq, sciATAC-seq, and scTHS-seq [78] (Figure 1B and Table 2). 

### 3.1. Epigenomics from Single Cells

Human DNA wraps into nucleosomes, which then condense into solenoids, forming the chromatin that comprises chromosomes. To assist in the discovery of chromatin modulators and regulators, the assay for transposase-accessible chromatin using sequencing (ATAC-Seq) was developed, first in bulk and now in single cells [71,79,80,81,82]. Recently, the approach was applied to joint measurements in the same cell [83,84,85]. In general, ATAC-seq measures the openness of DNA, a proxy for how easily transcription factors or other regulatory elements may bind. ATAC-seq is based on the use of a hyperactive Tn5 transposase that binds and cleaves open DNA before adding adaptors that tag accessible chromatin [79]. The identification of accessible DNA, or peak calling, is the core analysis function of ATAC-seq [86]. Gene regulatory assessments follow from peak calling to include differential peak analysis across cell types or experimental conditions, motif identification, nucleosome positioning, and transcription-factor footprint occupancy. The goal of these analyses is to infer gene regulatory networks and identify their key regulators. Analytical tools specifically designed for scATAC-seq datasets either with or without scRNAseq, including chromeVAR, Signac, and MAESTRO, were developed, although computational challenges remain [87,88,89,90,91].

To study HIV-1 latency, epigenetic profiles generated by ATAC-seq could be used to identify noncoding regulatory elements that associate with the transcriptome changes of CD4^+^ T-cell activation and/or HIV-1 latency reversal. At a minimum, multimodal interrogations of latency models can establish the relationship between epigenome and transcriptome during latency reversal. To facilitate this work in the context of HIV-1, RNA-seq and ATAC-seq datasets in bulk and single HIV-uninfected primary CD4^+^ T cells and their phenotypic subsets were published, providing a useful comparator framework to evaluate the specificity of observed changes in the presence of integrated HIV-1 [92,93,94,95,96].

### 3.2. Integrated Protein and RNA Measurements 

The development of methods to simultaneously quantify surface-protein expression and mRNA transcription is a seminal advance in droplet-based single-cell sequencing techniques. These approaches digitize the abundance of cell-surface proteins into discrete sequence information that can be coupled to and analyzed along with transcriptome measurements in single-cell droplets. Monoclonal antibodies (mAb) that would traditionally be conjugated to fluorophores or metals for flow or mass cytometry are instead labelled with DNA barcodes. The multiplexed identification of surface proteins using barcodes significantly exceeds the spectral capacities of flow cytometry or the number of available metal isotopes; DNA-barcoded mAb panels that simultaneously interrogate all known cell-surface proteins have been commercialized. Two studies that were initially described in 2017, using similar methodologies, explored these approaches. Cellular Indexing of Transcriptomes and Epitopes by Sequencing or CITE-seq detects multiple proteins and simultaneously characterizes the unbiased transcriptomes at the single-cell level [74]. Qualitative and quantitative comparisons of CITE-seq and flow cytometry in this report demonstrate similar results in PBMC. To the best of our knowledge, a comparison of CITE-seq and mass cytometry has not yet been reported. The RNA expression and protein sequencing assay or REAP-seq analyzes protein and RNA levels in single cells using DNA-conjugated antibodies and droplet microfluidics [75]. Both REAP-seq and CITE-seq leverage the difference in size between amplified cDNAs and antibody-derived tags to generate independent sequencing libraries. REAP-seq was benchmarked against flow cytometry for the detection of B lymphocytes, T lymphocytes, natural-killer (NK) cells, and monocytes, and delivered comparable results. 

The CITE-seq approach was recently extended to an expanded CRISPR-compatible cellular indexing of transcriptomes and epitopes by sequencing (ECCITE-seq), an approach that was adapted to 5′ capture-based scRNA-seq methods [97]. ECCITE-seq was used to perform CRISPR screens, reliably identify and quantify the heterogeneity present in single cells that received the same guide RNA (gRNA), and further define the mechanisms that control programmed death-ligand 1 (PD-L1) expression [98]. CITE-seq was applied to profile and compare HIV-1 infected cells during immediate and delayed antiretroviral therapy [99]. Single-nucleus-based CITE-seq, Intranuclear Cellular Indexing of Transcriptomes and Epitopes (inCITE-seq), was reported that measures quantitative intranuclear protein levels and transcriptomes in cells and tissues [100]. 

### 3.3. Other Frontier Single-Cell Technologies 

Some recently described approaches may hold promise for future studies of HIV-1 latency. Split-pool ligation-based transcriptome sequencing (SPLiT-seq) labels individual transcriptomes from cells or nuclei by combinatorial barcoding, generating uniquely barcoded cells; therefore, SPLiT-seq does not require cell partitioning workflows such as cell sorting, custom microfluidics, and microwells [101]. Taking advantages of scRNA-seq, single-nucleus RNA-sequencing (snRNA-seq) was introduced to visualize the extremely low levels of mRNA in a single nucleus, including sNuc-DropSeq [102] and DroNc-seq [103,104]. snRNA-seq is widely applied to recapitulate transcriptome and gene expression responding to a certain stimulus in neuronal cells and tissues [105]. However, primary cells used for HIV-1 latency investigation are rich in proteases and RNases [106], which may limit the application of these approaches in HIV-1 latency research. 

**Table 2:** Single-cell techniques.

Single-Cell Method	Acronym	Target	Reference
Single-cell RNA sequencing	scRNA-seq	mRNA	[107]
Switch mechanism at the 5′ End of RNA templates single-cell sequencing	SMART-seq	Full-length capture of RNA	[108]
Massively parallel single-cell RNA-sequencing	MARS-seq	3′-end only	[109]
Drop-seq	Drop-seq	3′-end only	[110]
Indexing droplets RNA sequencing	InDrop	3′-end only	[111]
Single-nucleus RNA sequencing	snRNA-seq	RNA	[112]
Massively parallel single-nucleus RNA-seq	DroNc-seq	3′-end only	[103]
Single-cell combinatorial indexing RNA sequencing	Sci-RNA-seq	3′-end only	[113]
Split-pool ligation-based transcriptome sequencing	SPLiT-seq	3′-end only	[101]
single-cell assay for transposase-accessible chromatin sequencing	scATAC-seq	Chromatin accessibility	[79]
Chromosome conformation capture (3C) coupled with sequencing	Hi-C/3C-Seq/Capture-C	Chromatin structure	[114]
Droplet-based single-cell chromatin immune-precipitation sequencing	scChIP-seq/Drop-ChIP	Chromatin fragments	[115]
Single-cell transposome hypersensitive site sequencing	scTHS-seq	Chromatin accessibility	[116]
Chromosome conformation capture sequencing combining chromatin immunoprecipitation	HiChIP	Chromasome capture	[117]
Single-cell combinatorial indexing ATAC-seq	sciATAC-seq	Chromatin accessibility	[118]
Cellular indexing of transcriptomes and epitopes by sequencing	CITE-seq	Multiomic	[74]
RNA expression and protein sequencing assay	REAP-seq	Multiomic	[75]
Expanded CRISPR-compatible cellular indexing of transcriptomes and epitopes by sequencing	ECCITE-seq	Multiomic	[97]
Intranuclear cellular indexing of transcriptomes and epitopes	inCITE-seq	Intranuclear protein and transcriptome	[100]

## 4. Integration of Single-Cell Datasets 

Single-cell high-throughput techniques characterize cell and tissue heterogeneity, and cell phenotype, fate, and function in a high-resolution manner. To increase the power of these datasets, single-cell sequencing data generated across different conditions, experiments, and samples could be integrated. With the rapid progression of machine-learning techniques, computational efforts focus on the integration of multiple sequencing modalities, including the integration of scRNA-seq data from multiple scRNA-seq experiments using different methods, the classification of multiple cell phenotypes from different studies, and joint analysis of transcriptional and spatial signals. The combination of multiple datasets generated by different high-throughput single-cell methods requires new computational methods that can integrate multiple types of single-cell data, detect correlations, and reveal relationships across modalities. While the single-cell genomic field continues to rapidly develop and evolve, we summarize some common and high-impact analysis strategies for these complex datasets. The discussed toolkits in this section are either in current widespread use in the field or have particular promise to advance the analysis of single-cell datasets in unique ways.

### 4.1. M3S

Many statistical methods that leverage Poisson (P), negative binomial (NB), and Gaussian (G) distribution approaches were developed to integrate scRNA-seq datasets generated from different conditions or experimental platforms [119]. Multimodal Model Selection (M3S) is an R package that selects the most parsimonious model to fit the distribution of gene expression in a genewise manner, characterizes the transcriptional events, and detects differentially expressed genes for scRNA-seq data.

### 4.2. Mixscape 

Other approaches were developed to integrate datasets in multimodal single-cell screens. With the use of targeting guide RNAs (gRNAs) in ECCITE-seq, heterogeneities with no perturbation effects present high background noise into downstream analyses. The Satija group designed a novel computational method, mixscape, for reducing such noise and refining the characterization of multimodal perturbations and relevant transcript and surface-protein levels. Heterogeneity was observed in the independent analysis of control cells with nontargeting gRNAs (NT cells). To obtain the transcriptome that revealed the real genetic perturbation, the most similar mRNA expression profiles of NT were subtracted from the original RNA profiles of target cells. 

On the basis of the Mixture Discriminant Analysis (MDA) algorithm, ECCIT-seq grouped cells on the basis of their expression of gRNA. Each group had a mixture of perturbed and nonperturbed subpopulations. The Gaussian mixture model was used to assign data points in each group on the basis of their subclass identity. The nonperturbed group had similar perturbation to that of NT cells. Mixscape is capable of grouping cells to perturbed or nonperturbed group [98].

### 4.3. Seurat Toolkits

Originally developed as a computational method to generate spatial transcriptomes in zebrafish embryos, Seurat is an R package that is in widespread use to analyze scRNA-seq datasets and identify rare cellular subpopulations [120]. Subsequent versions of Seurat introduced strategies to integrate and identify cellular populations across multiple scRNA-seq datasets [56] and eventually extend these approaches to other modalities, such as scATAC-seq [121]. Most recently, Seurat version 4.0 incorporated a weighted nearest-neighbor statistical-analysis approach to analyze multimodal single-cell datasets and created Azimuth, a free web application that allows for users to map their datasets to a multimodal reference atlas that incorporates scRNA-seq and CITE-seq data [87]. Reference atlases are available for the peripheral blood immune system and some tissue types. In the context of HIV-1 research, Azimuth may be used to annotate previously published scRNAseq datasets, which did not simultaneously assess protein expression, with surface-protein expression information, particularly valuable in the context of CD4^+^ T-cell subsets that are notoriously similar in RNA transcriptomes. However, its operating characteristics in this regard require careful validation, particularly in the context of memory T-cell subsets and rare cell populations, e.g., stem-cell memory cells, which may be important in HIV-1 reservoir dynamics.

### 4.4. LIGER

The main challenge of computational tools for the flexible modeling of single-cell datasets is the immense data heterogeneity generated from multiple modalities. A recently introduced statistical algorithm, Linked Inference of Genomic Experimental Relationships (LIGER), identifies shared cell types across samples. LIGER integrates in situ and gene-expression data to reveal the accurate spatial location of subtypes of cells, and jointly defines cell types by coupled scRNA-seq and DNA methylation profiles, providing a tool to characterize epigenomic regulation in specific cell types. After a common process of a raw digital gene-expression (DGE) matrix and dataset normalization, LIGER generates a dimensionally reduced space by integrative non-negative matrix factorization (iNMF) [122,123], and bases joint clustering on a shared factor neighborhood network showing cells with similar factor-loading patterns [122]. 

### 4.5. Harmony

Recent work introduced a flexible and scalable algorithm across datasets, Harmony, to integrate datasets that have experimental, biological, and technical differences [124]. Harmony uses an unsupervised scRNA-seq joint embedding strategy that projects cells into a common low-dimensional space. Cells in Harmony are clustered by a soft k-means algorithm instead of a specific condition or platform. Clusters of small subsets of datasets are penalized by an information theoretic metric. Multiple penalties are allowed for in Harmony, and datasets from different techniques or sample sources are thereby accommodated with soft clustering. Harmony creates the final cell cluster after several iterations of adjustments on the basis of the cell-specific linear factor and is available as part of established pipelines in several analytical packages. 

### 4.6. BindSC

As the number of single-cell techniques increases, it is increasingly common that, while these analyses are performed on the same sample, they do not simultaneously interrogate all features from the same cell. Therefore, improved computational methods are needed to integrate the data matrices that each technology, e.g., scRNA-seq, scATAC-seq, CITE-seq, and spatial transcriptomics, generates, and align these features being characterized across cells. To generate accurate coembedding matrices from multimodal data, a computational data-integration toolkit called biorder integration of single-cell data (bindSC) was recently developed to integrate variant single-cell data [125]. On the basis of biorder canonical correlation analysis (bi-CCA), bindSC accurately identifies the simultaneous alignment of columns and rows between data matrices from multimodal technologies, such as scRNA-seq and scATAC-seq, on the same biological sample. The bindSC method was tested for its ability to integrate different types of datasets to include epigenomic and transcriptomic, scRNA-seq, and spatial-transcriptomic data, as well as scRNA-seq and protein data. BindSC initially preprocesses individual datasets by selecting variable features and clustering cells, matching the initial feature across modalities, identifying correspondence on the basis of the bi-CCA algorithm, integrating clustered cells in a latent space, and constructing multiomic profiles.

### 4.7. MAESTRO

To integrate data from scRNA-seq and scATAC-seq datasets, the Shirley Liu lab developed the Model-based AnalysEs of Transcriptome and RegulOme (MAESTRO) workflow [89]. MAESTRO provides multiple functions to characterize the transcriptome and regulome, including cell-cluster integration and automated cell-type annotation. MAESTRO is compatible with multiple scRNA-seq and scATAC-seq platforms and inputs of different sequence files that are generated across these platforms. The creators presented data that MAESTRO may perform statistically significantly better than SnapATAC, cicero, and Seurat can in some aspects of scATAC-seq and scRNA-seq dataset integration.

### 4.8. ScAI

Another approach to address the limitation that data integration of parallel RNA-seq and ATAC-seq is asymmetric, relying more on scRNA-seq data, is the single-cell aggregation and integration (scAI) method [126]. scAI uses an unsupervised iterative-learning approach and, in the simulation modeling of three existing datasets, was shown to effectively integrate epigenomic and transcriptomic datasets. Some data were also presented that scAI may offer advantages when different cell types are coassayed. 

## 5. Conclusions

Single-cell sequencing approaches hold great promise to identify mechanisms that control viral latency reversal and define whether a cellular program associates with HIV-1 reactivation and under which cellular conditions. Recent advances in high-throughput techniques enable the integration of multiple single-cell methods, simultaneously profiling gene expression, epigenetics, and surface and intracellular protein content. Multimodal analyses are required to generate novel insights into the biological diversity and cellular heterogeneity that underlies HIV-1 latency and the regulatory networks that govern its reversal. While benchmarking studies are yet to reveal a consensus approach to the computational analysis of single-cell datasets, the development of a unified approach among HIV researchers should be considered to be a high priority. In the meantime, investigators that tailor their individual methods to their systems’ biology and the data types they generate may help to most expeditiously explore this new research space and lay the foundation for a future consensus on an integrated single-cell analysis pipeline. 

## Figures and Tables

**Table 1:** Published single cell studies of HIV-1 latency reversal.

Study	CD4^+^ T-Cell Source	HIV-1 Source	Studied Cells	Latency Model	Studied LRA	Single-Cell Approach
Golumbeanu et al.	HIV-donors Two treated, suppressed participants with HIV	VSV-G pseudotyped NL4-3/GFP reporter deleted in *gag*, *vif*, *vpr*, *vpu*, *env*, and *nef* (pNL43-Δ6-dreGFP) Participants’ HIV-1 isolates	Cocultured CD4^+^ T cells negative magnetic immunoselection of CD25^−^CD69^−^HLA-DR^−^ CD4^+^ T cells	H80 feeder model, 8-week culture N/A	Vorinostat 500nM αCD3/αCD28 beads αCD3/αCD28 beads	SMART-Seq
Bradley et al.	HIV donors	CXCR4-using pNL43-Δ6-dreGFP	Cocultured CD4^+^ T cells	H80 feeder model, 8-week culture	αCD3/αCD28 beads with lowest 15% GFP-expressing cells only	3′ 10× Genomics
Cohn et al.	Three treated, suppressed participants with HIV	Participants’ HIV-1 isolates	Env^+^Gag^+^ cells obtained after 36 hr PHA activation + pancaspase inhibitor and enrichment with 3BNC117/10-1074/PG16 bnAbs	N/A	PHA/IL-2, 36 h incubation	SMART-Seq
Liu et al.	Fourteen treated, suppressed participants with HIV	Participants’ HIV-1 isolates	CD4^+^ T cells probe-positive for 5′ and 3′ HIV-1 RNA (SortSeq)	N/A	PMA/ionomycin, 16 h incubation	SMART-Seq

## Data Availability

Not applicable.
